# One Health: a concept led by Africa, with global benefits

**DOI:** 10.1136/vr.h2461

**Published:** 2015-05-09

**Authors:** Titus Mlengeya Kamani, Rudovick Kazwala, Sayoki Mfinanga, Dan Haydon, Julius Keyyu, Felix Lankester, Joram Buza

**Affiliations:** Minister of Livestock and Fisheries Development, Government of the Republic of Tanzania, Mandera Road, Dar es Salaam, Tanzania; Faculty of Veterinary Medicine, Sokoine University of Agriculture, Morogoro, Tanzania; Muhimbili Research Centre, National Medical Research Institute, Dar es Salaam, Tanzania; Boyd Orr Centre for Population and Ecosystem Health, Institute of Biodiversity, Animal Health and Comparative Medicine, University of Glasgow, Glasgow; Tanzanian Wildlife Research Institute, Arusha, Tanzania; Paul G. Allen School for Global Animal Health, Washington State University, Pullman, WA 99164, USA; School of Life Sciences and Bioengineering, Nelson Mandela African Institution of Science & Technology, Arusha, Tanzania

## Abstract

Titus Mlengeya Kamani and others argue that Africa is well positioned and equipped to conduct and benefit from an integrated approach

ONE Health evolved from the recognition that an interdisciplinary approach is required to understand complex health problems, and that the health of humans and animals are inextricably linked. Through closer cooperation between the human, veterinary and environmental health sectors, added value, in terms of health metrics, cost savings and environmental services is achievable. Although the One Health concept has been recognised for many years, particularly since the seminal work of Calvin Schwabe (Schwabe 1984), many challenges remain in making it operational.[Fig VETRECG3917F1]

‘Africa's National medical and veterinary institutions are still maturing, which presents African health professionals with an opportunity to build on an instinctive understanding of the connectivity between people, animals and their environments, and to “leapfrog” barriers imposed by more well-established and rigid institutional systems’

**Figure VETRECG3917F1:**
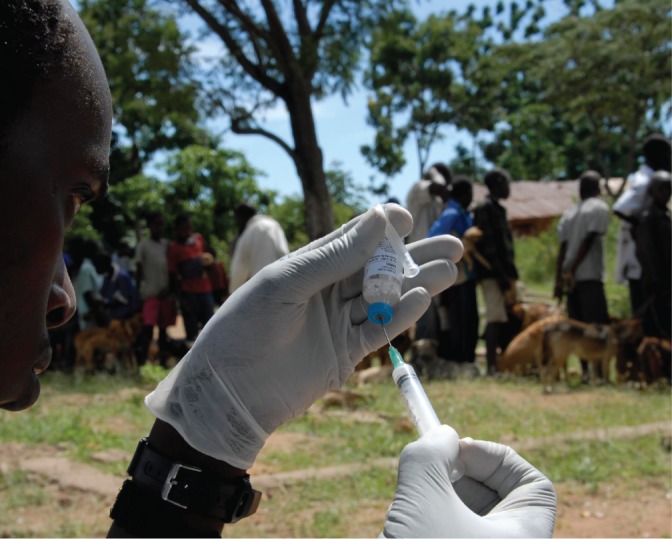
A vaccine is prepared while owners of domestic dogs in a village in northern Tanzania wait in line for their dogs to be immunised against rabies

Image: Felix Lankester

Following a succession of global disease problems, such as highly pathogenic avian influenza, SARS, Ebola virus disease and bovine spongiform encephalopathy, which all had their origins in animal populations and are linked with agroecological change, it is perhaps surprising that One Health has gained so little mainstream traction among biomedical professions. A possible explanation is that separate animal and human health agencies, responsible for disease prediction, prevention and control, have been embedded in many developed countries since the 19th century, with institutional barriers impeding horizontal collaboration. The resulting gulf between human and animal health, caused by disciplinary conventions and cultures rather than scientific rationale, divides medicine in two.

In Africa, however, where people's lives are intimately related to the health and productivity of livestock and the natural environment, the situation is different. National medical and veterinary institutions are still maturing, which presents African health professionals with an opportunity to build on an instinctive understanding of the connectivity between people, animals and their environments, and to ‘leapfrog’ barriers imposed by more well-established and rigid institutional systems. If this succeeds, African scientists and African institutions have the opportunity to become world leaders in One Health. The establishment of an effective inter-ministerial zoonotic disease unit in Kenya, which has rapidly developed integrated national plans for rabies control and elimination, is one example of how these opportunities are being met with innovative cross-sectoral structures.

Opportunities for One Health in Africa are also provided by new academic organisations, such as the Nelson Mandela African Institutions of Science and Technology (NMAIST), which aim to train and nurture the next generation of African scientists to address the development needs of the continent. These fledgling institutions – the Tanzanian campus of NMAIST opened its doors in 2010 – are nimble and unencumbered by history, and provide African-based research scientists novel opportunities for integrated research. For example, the science of One Health is now formally integrated as part of the Arusha NMAIST School of Life Sciences and Bioengineering curriculum.

Several examples of the benefits of a One Health approach have been demonstrated within an African context. For example, collaborative projects implementing joint livestock and child vaccination campaigns in pastoralist communities in Chad have resulted in economic savings for the Chadian public health and animal health Ministries and, importantly, improved vaccination coverage of children and women who would otherwise have no access to healthcare (Bechir and others 2004, Schelling and others 2007, Zinsstag and others 2012). Integrated human and animal zoonotic disease surveillance in Ethiopia has identified epidemiological links between bovine TB in humans and animals (Gumi and others 2012, Greter and others 2014). Shared human and veterinary laboratories to diagnose brucellosis in febrile patients in Mali have resulted in brucellosis being considered as a differential diagnosis for febrile illness (along with malaria and typhoid fever) in an area where raw milk consumption is still prevalent (Steinmann and others 2005, Zinsstag and others 2012). Such outputs of One Health programmes are normalising the practice of professionals from all relevant disciplines working together. In Tanzania, we have a long history of One Health integration across disciplines, with researchers from the Sokoine University of Agriculture, the National Institute of Medical Research and the Tanzania Wildlife Research Institute, having conducted joint programmes on bovine tuberculosis, brucellosis and rabies for over 20 years.

Another exciting new international development that will benefit the next generation of African scientists is the Alliance for Accelerating Excellence in Science in Africa (AESA), set up by funding from the Wellcome Trust, DFID and the Bill and Melinda Gates Foundation (Nordling, 2015). Historically, with a large proportion of Africa's scientific research financed by funds coming from western Europe and America, its research agenda has been set by priorities from outside the continent. The development of this Africa-based platform, however, will finally enable international research funding to be based within Africa where Africans can manage it. This will enable African-based scientists to set their own research agendas that answer the questions that are of most significance to the continent.

For such programmes to become mainstream, added value above that achieved by human and animal sectors working alone must be demonstrated. A key component of this will be the development of synergistic international partnerships that fuse the technological potential of industrialised nations with the still more flexible research and political cultures present in Africa. Existing initiatives, such as the Zoonoses and Emerging Livestock Systems (ZELS) (a consortium of UK funders including DFID and the BBSRC) and the Afrique One programme (funded by the Wellcome Trust), which bring together African-led multidisciplinary research teams from both East and West Africa to tackle some of the continent's most complex health issues, are examples of how such partnerships can work.

‘The world is looking to African researchers as world leaders to develop the emerging discipline of One Health, and the health of the global community will undoubtedly benefit from its effective implementation’

With high rates of globalisation, urbanisation and fragmentation of wilderness areas, the emergence of novel diseases that affect humans and animals alike is set to increase. To understand and mitigate these disease problems, a One Health approach, that fosters closer cooperation between human, animal and ecological health, must become mainstream. The outcomes, such as the early detection of disease or the implementation of appropriate control measures, will potentially result in economic savings running into the billions.

The rich diversity of African landscapes and the variety of livestock-keeping practices, together with concerns such as increasing migration flows and food insecurity, mean that African institutions and African scientists are well positioned and equipped to conduct, and benefit from, this integrated research that is of clear importance to Africa. It is also African scientists and African policymakers who have the greatest opportunity and responsibility to take leadership of One Health and develop the integrative institutional frameworks and research programmes needed to tackle these complex health problems. The world is looking to African researchers as world leaders to develop the emerging discipline of One Health, and the health of the global community will undoubtedly benefit from its effective implementation.
